# Observations on Scarlet Fever, as It Appeared in Steubenville, Ohio, during the Winter of 1831 and 1832

**Published:** 1833

**Authors:** A. Judkins


ART. II.—*Observations on Scarlet Fever, as it appeared in Steubenville, Ohio, during the winter of* 1831 *and* 1832. By A. JUDKINS, M. D.

During the fall and early part of the winter of '31 and '32, a few sporadic cases of scarlet fever occurred in this town, but they were comparatively mild, and yielded readily to remedies. About the first of January, 1832, the disease assumed the character of an epidemic, which continued to rage with great severity, until about the middle of March, when it abated, but did not disappear until midsummer. The month of June was very warm, nevertheless the disease was decidedly worse during *this* than the two preceding months; some of the worst cases occurred in this month. The number of deaths from the disease is not precisely known, but supposed to be about eighty—our entire population is about three thousand.

The disease had prevailed in some of the neighboring towns and districts for two or three years, before it visited Steubenville; within which time it had almost surrounded us. More than a year before its appearance here, it had prevailed in districts on either side and not more than fifteen miles distant, and wherever it did prevail, was attended with great mortality. The first deaths here were in the latter part of December. In the first week of January, I lost two patients in the same family; they died within two days of each other, one in twenty-two, and the other in thirty-six hours from the attack. I was surprised by the suddenness of death in these cases, but was forced to ascribe it to cerebral congestion, from the symptoms of the disease, and time, and manner of death. The body examined, as reported at the end of this paper, was that of the last of these. In the first of these cases, there was no blood drawn; in the second but a very small quantity, and in neither of them was there any effectual operation from cathartic medicine.

I must be permitted to say, that the division of this disease as found in the books, is altogether unsatisfactory—it was not always possible to recognize the peculiarities, which are said to mark its different varieties. A more natural division of it would seem to be, into simple and congestive, or compound scarlet fever, inasmuch as this will enable us to distinguish those cases, which, from their mild and lenient character, scarcely indicate the use of remedial agents, from those in which the utmost attention is frequently necessary to avert a fatal issue; and to point us to the real source of danger.

Dr. Sydenham has said, that simple scarlet fever, "is but a disease in *name*, and only dangerous from the too great officiousness of the physician." It is certainly a mild disease, and one in which medicine need scarcely be given. We had many cases of it during the late Epidemic, and the only consideration calculated to attach importance to it, is the danger always to be apprehended, of its changing its character, and assuming the congestive form. The symptoms which characterize simple scarlet fever, are well described in our books—they are those which, in general terms, indicate very moderate febrile excitement; if preceded by a chill, it is apt to escape observation. Sometimes the fever will be considerable, but superficial, seeming to expend itself mainly upon the surface; some soreness of the throat attends, the patient complains of weakness, and of uneasiness in the head; pulse never exceeding twenty beats in the minute, unless the child be very young and irritable. In two or three days, the eruption is apt to make its appearance, which in due time disappears, followed by desquamation. During the presence of this form of the disease, the stomach and bowels are somewhat deranged, vomiting rarely attends—evacuations from the bowels, frequently dark. As I have said before, little need be done. It is proper to protect the patient from cold, and preserve the bowels open by the use of gentle cathartics, such as castor oil, magnesia, or rhubarb. But slight and unimportant as the disease may be, the physician should bear in mind, that there is always danger of its assuming a different and more dangerous aspect; I saw many cases in which the symptoms for two or three, and in one instance, four days, were so mild as scarcely to require medicine, suddenly, and without any assignable cause, assume the most alarming character.

*Congestive or complicated Scarlet Fever*, is a very formidable disease; consisting of the usual symptoms of scarlatina, to which local congestion is super-added. Perhaps it may be said, that local *inflammation* may be present; this may be true, as I am not able to prove the contrary, and therefore I have designated this form of disease congestive, or *complicated*. I am strongly impressed with the opinion, however, that, in scarlet fever, when any of the viscera of the body are involved in disease, venous congestion and not inflammation, forms the general character of the local affection. Having been present at but one dissection of a body after death from the disease, I could not rely upon the evidence, however strong, which that case afforded, of the truth of my opinion; but upon the fact, rather, that during the entire period of our epidemic, although complicated cases of the disease presented themselves constantly, affecting, in turn, most of the viscera of the body—yet the phenomena of inflammation of any of these viscera were not observed in a single instance. This fact, if one it be, is of no particular practical importance, although it may serve to explain, in some instances, the effect of remedies.

The organs most frequently involved in this congestion, were the *Brain*, *Lungs*, *Stomach*, *Bowels*, and *Liver*. The disease of the throat was always present; this, although rarely the source of danger, will be described hereafter.

*Scarlatina with Cerebral Congestion.* It has frequently been remarked, that inasmuch as the brain, in childhood, is more perfectly developed than the other viscera, it is more obnoxious to disease, a priori, than any other. Whether it be attributable to this, or some other cause, it is certain, that in a large majority of cases of the late epidemic here, the brain was the organ more especially involved. The attack was usually sudden, and generally ushered in by vomiting, which was followed by speedy and violent reaction, the pulse sometimes in a single hour from the attack, beating 150 strokes in the minнte; pain in the head was sometimes complained of, but not always, even when the patient was old enough to describe his sensations; the skin of the head and trunk preternaturally warm, while the extremities were generally cold; the tongue never failed to exhibit its peculiar appearance, it became furred and the papilla elevated; in one case, these were so much elongated that they might have been shorn with common scissors. This case terminated fatally on the fourteenth day. In some protracted cases the tongue became clean and dry—the throat was uniformly inflamed in a greater or less degree, usually exhibiting the specks or ulcers which accompany scarlatina anginosa; the pulse was always frequent in dangerous cases, above 150, sometimes 200 beats in the minute. In every variety of the disease, the frequency of the pulse afforded a good ground of prognosis. If it ranged at or below 130, a favorable issue might be anticipated; this rule was rarely deceptive: bowels uniformly torpid. The eruption was by no means uniform in the time of its appearance, sometimes it would shew itself on the first, and sometimes as late as the third or fourth day, and in some cases it was absent entirely. If in this form of the disease, active remedies were not used early, the case never failed to terminate fatally;—every thing depended upon this, and where the disease was permitted to run its course unchecked, even for a few hours, the issue was always doubtful,—and if it did not terminate fatally in two or three days, it made an impression upon the constitution, and especially upon the brain, from which the patient could scarcely recover. Two cases came under my observation, in which the attacks were violent, accompanied from the first with great determination of blood to the brain, threatening the speedy extinction of life; the treatment was rather temporising—they were bled in small quantites; the treatment served to avert fatal congestion for a time, but it was inadequate to the cure of the disease; they were protracted, one to thirteen and the other to fourteen days, when they terminated fatally. During the entire period, evidence of cerebral disease was very clear, and death at last, was obviously the result of this. Sometimes the attack was less violent; for two, three, or four days the fever would be moderate and diffuse, the brain scarcely seeming to suffer, when after the lapse of several days perhaps, the heat of the head, throbbing of the carotid and temporal arteries, with suffusion of the eyes, would clearly indicate cerebral disease. During the month of February, especially, many cases occurred in which the force of the disease fell upon the *lungs*. In these cases the attack was not usually so sudden, and so far as my experience enables me to say, was not so frequently accompanied with vomiting. Here, a cough, and the usual symptoms of pulmonary irritation were superadded to the other symptoms of scarlatina: the cough was usually dry in the early stage, and indeed, until a crisis was attained. I had one patient, a little boy two years old, in whom this cough was almost incessant for two weeks; he was in charge of a most vigilant nurse, and she assured me that there was no time during this period in which he had half an hours respite from it. In this case the pulse for fifteen days was constantly 160 or more, in frequency. He recovered. The pulse, in this form of scarlatina, was quite as frequent, as in cases accompanied with cerebral congestion; the bowels were less torpid, medicine operating upon them with less difficulty: the appearance of the throat and tongue not differing, so far as noted from the variety above mentioned.

The appearances of the throat were various; I am not aware that this ever escaped entirely, but the inflammation in some dangerous cases was very trifling; in others, there was, very early in the disease, general redness of the face, approaching in some cases to a purple hue, accompanied with ulcerated specks on the glands especially, but occasionally on different parts of the fauces and palate. A singular circumstance was occasionally noticed, as connected with this inflamation of the throat, but which has not escaped the observation of authors,—it is, that although much inflamed and ulcerated, the patient is unconscious of it. I saw a few cases of this, the first was a lady who took the disease a few days after her confinement in childbed. The symptoms of scarlatina were clearly defined, but she insisted that she had no soreness, or other uneasiness of the throat; the next day, however, on examination, her fauces were found of a deep scarlet hue, with many deep seated ulcers of the tonsils. A few cases occurred in which the tonsils were so much swollen as to endanger suffocation; they suppurated and discharged large quantities of pus: these cases were confined, so far as I know to adults. A stiffness of the muscles of the neck, rendering the motion of the head painful was frequently observed.

The lining membrane of the mouth and throat presented, in some cases, one entire ulcerated surface accompanied with dry ulcers of the lips and nose, and many of the symptoms of scarlatina maligna. This state was preceded, it is believed in every instance, by symptoms of high excitement, which lasted several days. A little boy, six years old, was attacked suddenly and violently, with the symptoms above mentioned as characterizing scarlatina with cerebral determination. He was bled and cupped freely, took an emetic, and used the other remedies to be mentioned hereafter for this form of the disease, and on the fourth day all danger seemed to have passed off, he was, apparently convalescent: His throat had been considerably inflamed, but not unusually so; but at this time, however, it was observed that his mouth and throat were about to assume the ulcerated appearance—and in twenty-four hours the lining membrane presented one entire ulcerated surface. The orifice from bleeding had healed, but it became open, and assuming a species of dry ulceration, continued to enlarge for several days—a low typhoid fever attended, accompanied with an irritable state of the stomach and bowels, with occasional vomiting and purging—His life was almost despaired of for upwards of two weeks, but he finally recovered after the lapse of seven weeks. A suspended secretion of urine attended in this case much of the time. Anasarca and ascites, with temporary deafness supervened—but he is now entirely recovered from them, and enjoys good health. This form of the disease had many of the attributes of cynancha maligna; it was attended by a low typhoid fever, frequent and quick pulse, dry skin, with all the signs of adynamia. If the patient had been bled, the orifice would either not heal, or if healed, would open, and assuming an unhealthy character, perfectly dry, would enlarge from day to day, which was never arrested until the death of the patient, or a favorable crisis of the disease. I knew of but one recovery after this state of the skin occurred. The extent of this sloughing, or ulceration, was frequently very great. I saw one case in which the skin was destroyed for several inches, leaving the vessels and tendons bared; the patient was much exhausted from the low typhoid fever, which attended, and which was accompanied with delirium, and some twenty days of suffering, was suddenly cut off by hemorrhage from the wound, occasioned by one of the vessels being involved in the process of destruction. Mr. Colden alludes to this state of the skin, when he says, in reference to the impropriety of depletion in cynanche maligna, "the consequence of evacuations is an insurmountable tendency to mortification, so that the very orifice made by the lancet mortifies." It is proper to remark, however, that this state of the skin, although it is believed to have attended in every case of the disease in which the ulcerated state of the mouth just mentioned was present, was not, by any means confined to them. The train of symptoms above mentioned, as constituting a close approximation to scarlatina maligna, or cynanche maligna, did not occur very frequently; but the condition of the skin just mentioned, as evidenced by the state of the orifice from bleeding, occurred, so far as I know, in every fatal case of scarlatina, when death did not take place within the first week. My attention was early directed to this fact; I examined the orifice made by the lancet, when the patient had been bled, with as much interest as I did the pulse, especially with a view to a prognosis. If the patient had been bled early in the disease, and the orifice healed, and the case terminated fatally at last, death in several cases is known to have been preceded by an opening of the orifice: but more commonly, the orifice would not heal, if the case was to terminate fatally. That the *bleeding* had any agency in the production of this state of things, cannot by any means be admitted, the reverse is believed rather to have been the truth.

The condition of the stomach, bowels, and liver, will be noticed in the course of the treatment.

The cause of scarlatina, whatever it may be, operates with peculiar force; the attack is usually sudden, and so violent, that unless active remedies are called in requisition early, the patient is lost. The first effect of the disease is to drive the circulation and excitement from the surface, and to concentrate them upon some vital organ; this impress may be so violent as to prostrate, in a single hour, the vital energies to such a degree, as to prevent the power of reaction. My friend, Dr. Andrews, had well nigh have lost a patient from this cause, but by the use of the warm bath and mustard to the surface, he succeeded in exciting reaction. Writers upon this disease, as well as some other epidemics, speak of sudden deaths occurring during their prevalence without any assignable cause. There can be little doubt, I think, that these sudden deaths are the effect of the first impress of the disease, so paralyzing the powers of the system, as to extinguish life immediately, without any of the usual phenomena of the disease. Reaction, however, usually follows, and follows very rapidly, and if this first paroxysm is not met by appropriate remedies, it will either extinguish life in one or two days, or produce such a degree of engorgement, or lesion of the organ involved, as to render all attempts to save the patient unavailing. I allude of course to the more severe form of the disease. The object of our remedies is to restore the lost balance of the circulation and nervous energy, and to protect the organ upon which the force of the disease seems to be expending itself. The remedies upon which reliance was principally placed, were: bleeding, general and local; emetics; cathartics; cold water; antimonials; blisters; &c.

*Blood-letting.* As the patient was rarely seen until reaction was established, if the pulse was frequent, as it always was, in bad cases, this remedy was usually resorted to early; and although it may have been used in some cases in which the cure might have been effected without it, and although success did not always follow its use, I am not advised that it was ever supposed to be, in any wise prejudicial. One thing is very certain, where this remedy was used freely early in the disease, the case never had the rapidly fatal termination, which did occur, where it was omitted. It would, at least, protract the disease, and give time to use other remedies, and this was the chief motive for its use. It was frequently necessary to repeat it, perhaps in a few hours, and sometimes on the second day, or even later in the disease. When the brain was the organ involved, the necessity for the remedy was more imperious. The quantity drawn, was not definite; but governed by the rules which direct the use of the remedy in other diseases. It is used here to prevent, say fatal congestion of the brain,—I might say apoplexy. I wish to guard the younger members of the profession, especially, against a doctrine as false as it is mischievous, that there is some peculiarity in the disease under consideration, which, if it does not deter us from bleeding altogether, should at least prevent us from being governed by the rules which govern us in the use of the remedy in all diseases. It was not usually carried to the extent of syncope. This is hardly safe with children in any disease, yet this did happen in a few cases, and without any bad consequences. The remedy was scarcely ever repeated more than twice, frequently it was not repeated at all; our object was at one bleeding, to take the quantity adequate to produce that impression upon the system, without which, bleeding, in acute diseases scarcely deserves the name of a remedy.

*Cupping.** This is a remedy of great value in scarlatina, and one upon which much reliance was placed in the treatment of the late epidemic. If the disease was accompanied with cerebral determination, the cups were applied to the temples, or the nape of the neck, or both. They are rarely calculated to supercede general bleeding; but when this had been premised, and the general tendency to congestion was still apparent, a few ounces of blood drawn from the temples or nape of the neck, exerted great influence over the disease. On the first attack, as much depended upon making an early impression upon the

* We have no medicinal leeches here.

disease, blood was frequently drawn from the neck or head, one or two hours after the general bleeding; cups draw blood more freely from the temples, than the back of the neck; from the former several ounces may be taken very conveniently. I have frequently produced a very sensible effect upon the pulse by typical bleeding from the temples, and thus the necessity ot repeating general bleeding is sometimes obviated. In the more advanced stages of the disease, the necessity for their use was very frequent. In some cases they were applied once or twice daily, for several days in succession. When the lungs were the seat of the disease, they were applied to different parts of the chest and back, with equal effect. I saw a few cases, especially in adults, in which the tonsils were so much swollen as to endanger suffocation; blood in these cases was drawn from the sides of the neck with the best results. I saw a young woman about twelve hours after her attack, insensible, with the glands of her throat so much swelled that she was unable to speak and scarcely able to breathe. I drew some blood from the arm, which induced vomiting, but she still refused to take drinks—waiting half an hour, and her breathing remaining the same, I drew perhaps two ounces of blood from the sides of the neck; when she was almost immediately able to swallow and breathe with comparative ease. I would impress upon the minds of physicians the importance of topical bleeding in scarlatina. Why it is that this remedy exerts more influence over visceral disease when the accompaniment of scarlatina, than when induced by other causes, may be a subject of speculation. If the fact exists, and it is believed it does, a knowledge of it is of the utmost importance in therapeutics.

*Emetics.* These have long been held in high estimation in the treatment of this disease, and their value does not seem to have been over-rated. There are two indications which they are intended to answer: in the early part of the disease, more especially, they are designed to equalize excitement, and restore the circulation to the surface, and thus to counteract the tendency to congestion; and in the malignant form of it they are prescribed for the purpose of removing from the stomach offensive matters, and to cleanse the throat. It was to fulfil the first of these indications almost exclusively, that they were used here. They usually constituted the first medicine given; if the patient was bled, the emetic was administered immediately arter, either alone or in conjunction with calomel. I usually gave the emetic singly, taking care to ensure its free operation before I administered the cathartic; it is of the first importance that the emetic should operate well, I preferred the tart. antimon. to any other article; if the child was under a year old, the wine of antimony was preferred. Governed by correct views of the object to be attained by emetics in this disease, Dr. Tully, of Albany, insists upon the superiority of the sanguinaria canadensis, over any other article. I confess that before the disease visited us, I felt inclined to join in this preference, but being more familiar with the use of other emetic articles, and finding the antimony produce what seemed to be the necessary effect, I was induced to adhere to it. Emetics were not confined to the early stage of the disease; if during its progress, the excitement seemed to be unequal, the tendency to congestion still present, they were repeated; they were frequently repeated more than once in the same case, under the same circumstances, and always with good effect. They were alike valuable whether the disease threatened congestion of the brain, or some other organ, and perhaps there was no remedy which exerted so much influence over the soreness of the throat.

*Cathartics.* If the experience afforded by the late epidemic can be relied on, one would be inclined to think that the importance of purging in scarlatina anginosa, has not been properly appreciated by the profession generally. Dr. Cullen merely recommends that the "belly be kept open." Dr. Rush, in speaking of the disease, as it appeared in Philadelphia in 1783 and 1784, says "During the whole course of the disease, where the calomel failed to open the bowels, I gave lenient purges, when a disposition to costiveness required them." Thomas recommends that you dislodge from the bowels, at the commencement of the disease, its feculent contents, and afterwards trust to injections. Underwood says, "Should the body be costive at the time of the attack, an opening medicine should be given previous to the administering of bark or cordials," and subsequently, "if the child should be two or three days without a stool, a laxative clyster should be injected." Gregory recommends moderate purging, and Dr. Calhoun, in his notes on Gregory's practice seems to contemplate nothing more than to keep the bowels in a soluble state. Now these authorities are much relied on; these books are to be found in almost every medical library in the country, and a physician may suppose that his collection of practical works is tolerably ample, when he may not be in possession of a single authority for *active purging* in scarlatina. It is true we are not without such authorities. Hamilton, Armstrong, Cazenave, Dewees, and some others, insist upon their use, not merely to obviate constipation of the bowels, but with a view to their purgative effect. Dr. Armstrong, who has shed more light upon the pathology of scarlatina, than any other man, says that "some judicious practitioners of my (his) acquaintance, entirely confide in tepid affusions and purgatives; and the great success which I have repeatedly witnessed from this treatment, warrants me in giving it a strong recommendation in this variety* of scarlatina."

No remedy may claim precedence of purging in scarlet fever. I believe that one of the earliest effects of the disease is, to suspend bilious excretion, and it is surprising how soon the bile in the bladder assumes a dark color. Until this is discharged, and the emunctories of the liver freely opened, the patient has little chance to get better; but so soon as this is accomplished, the improvement is almost uniform. I have known many cases in which mercurial cathartics operated freely in eight or ten hours from the first attack, and the stools invariably presented a dark and bilious appearance, in some cases having near the color and consistence of tar. Let us bear in mind the importance of effecting bilious evacuations from the bowels; no reliance could be placed upon the reports of nurses; perhaps the patient would pass *something* every few hours, but if its character was inspected, it would be found to be liquid, with, perhaps a small quantity

* Scarlatina Anginosa.

of the mucus of the bowels. The color of these dejections was various; sometimes tinged with green, frequently brown, or of a muddy color; but they were uniformly small in quantity, unless medicine produced its proper effect; whenever this could be attained, even the first day of the disease, the stools were either black, or of a dark green appearance, consistent, and in large quantities. Let the physician bear in mind, that he has no reason to expect the recovery of his scarlet fever patient, without obtaining the kind of stools last mentioned, and then there is little danger of his being deceived by the reports of nurses, or by his own observation. If he is not impressed with this important truth, even if he examines the alvine discharges ever so carefully, he may suppose that cathartic medicine has performed its office well, while, in fact, it has been utterly useless. The use of purgative medicines in our epidemic, was entered upon early: if they were not combined with, they were given immediately after the emetic. Calomel was always used, but to promote its operation, various other articles were given, such as castor oil, calcined magnesia, senna, croton oil, &c. To a child two years old ten grains of calomel, was frequently given at a single dose—sometimes it was given in small doses, frequently rejected, it became matter of much importance to find an article which would assist the calomel and set well upon the stomach. Croton oil was found to answer this purpose best: the following preparation was very effectual and much used:

℞. Calomel ℈j.

Croton oil, gts. iv.

Syrup simp. ℥j.

Dose, a teaspoonful every hour or two, adapted to a child two or three years old. When the stomach would retain it, castor oil was used instead of the syrup. Some other cathartic article was usually given in the interval. I gave to a child five years old, in thirty-six hours, one drachm of calomel, fourteen drops of croton oil, one ounce calcined magnesia, two ounces of castor oil, with perhaps a pint of strong senna tea; little or none of this was rejected. The child took injections very frequently at the same time; patient recovered. Mentioning this, as an extreme case, to one of my medical friends, he assured me that he had even exceeded it with one of his patients. If purgative medicine could be made to operate properly upon the bowels early in the disease, however severe the case, hopes of recovery might reasonably be entertained; for it not only served to mitigate the symptoms for the time, but tended greatly to facilitate their future operation. It was necessary to have purgative medicine operate daily, and the same attention to the character of the dejections, was always necessary. I think it is Armstrong who says that the operation of purgative medicine, even at the time the eruption is making its appearance, does not check it, or produce revulsion. I am rather inclined to believe that this is true, though, I confess, that having some doubts about it, I usually withheld them for a few hours after the eruption first appeared. No case is recollected in which the first appearance of the eruption was simultaneous with the free operation of cathartic medicine; but this was frequently effected a few hours after the eruption shewed itself, and before it had covered the body, and is not known to have checked, repelled, or in any degree interfered with it.

The *liver* always seemed to suffer. Only one dissection was made; in this, the appearance of the liver was scarcely changed, but the condition of the ducts, and especially the contents of the gall bladder, plainly indicated derangement of function. Calomel is believed to exert more influence over the function of the liver than any other article, it was therefore used, not exclusively, as a cathartic, until the disease was subdued, or the alvine evacuations indicated healthy action of the liver. Although calomel was used very freely every day, for several days in succession, sometimes more than a week, I am not sure that salivation occurred in a single instance. In one or two cases, the gums became swelled, but it is not certain that it was the effect of the calomel.

*Cold Water.* The frequency of cerebral congestion, led, very early to the use of cold applications to the head. Cloths dipped in water, bladders filled with ice, and the like were used; the hair being previously removed. But the most effectual way in which cold could be used was that suggested by Dr. Southwood Smith of London, and which he has named the "cold dash." Enveloping the head in ice, was evidently inefficient; it would keep the surface cool, to be sure, so long as it was on, but its removal was invariably followed by preternatural heat of the skin of the head, and it could scarcely be said to exert any permanently useful influence upon the disease of the brain. Under these circumstances the "dash" was called to our aid, and with the best effects. The manner of its application was somewhat different from that recommended by Dr. Smith, but we are indebted to him for the hint. The hair being removed by scissors, the patient's head was placed over a tub, or large basin, at the bed side, and with a pitcher or coffee pot, pour upon one particular part of the head, by a small continuous stream, from 1 to 2 gallons of cold water; ice water was used; the height from which it was poured was from two to four feet; the head was then replaced upon the pillow, and on becoming warm, the same was repeated. Perhaps for a time it would be necessary to repeat the "dash" several times in a single hour; sometimes it was repeated every ten or fifteen minutes for several hours together, and its influence in overcoming cerebral congestion was surprising. Dr. Cume proposes to extinguish cynancha maligna in the beginning by copious affusion of cold water; the same has been proposed in scarlatina anginosa. That this is sometimes practicable, from the use of the remedies above mentioned, aided by the "dash," there can be no doubt. I visited a little boy ten years old, in the evening, six hours after the attack; he was laboring under all the symptoms of scarlet fever, with cerebral congestion, and was comatose. He was bled, took an emetic of tart. antimony, which was succeeded by fifteen grains of calomel, and castor oil every three hours until free bilious evacuations were obtained from the bowels; the cold dash was also used, beginning an hour after the operation of the emetic. The "dash" was repeated during the night, perhaps thirty times, and in the morning the little fellow was sitting on the hearth cracking nuts. He scarcely complained afterwards, having no eruption. We generally waited to observe the effect of the emetic; if, as it sometimes happened, the skin became cool, accompanied with a gentle perspiration, the cold application was deemed unnecessary, and in many cases, the violence of the disease was arrested at this period, and proper attention to the bowels was only required to ensure the safety of the patient. The "dash" was used at any stage of the disease, when the determination to the brain was great; its effects were uniformly salutary. In very many cases, where the eruption was tardy in its appearance, this remedy was speedily followed by the most perfect scarlet efflorescence. I saw a patient who had labored under the disease for twenty-four hours; had not been bled, cathartic medicine had not produced the desired effect; the patient was entirely insensible; pulse perhaps 200; eyes suffused; extremities cold; could not be induced to swallow any thing. The cold dash and cold affusions were ordered, and synapisms to the extremities, and the case was trusted exclusively to this. In eight hours, when I again saw this patient, the body was about half covered with a deep scarlet eruption; pulse 140; patient sensible; tongue, which had been much loaded and dry, was now moist—in short, every symptom was much ameliorated. The cold water was ordered to be continued,—but what was my surprise, when on visiting this patient six hours after, and finding her as near as possible in the same situation as at my first visit, to learn that the "dash" and sponging had been discontinued since my last visit. The eruption which had come on a few hours after the first application of cold, had covered about one half of the body. On discontinuing the remedy, this disappeared, and the patient relapsed into her former insensibility, from which she never recovered. A high degree of visceral congestion seems to be incompatible with the appearance of the eruption, and the most effectual method of curing the disease, and indeed in bringing the eruption to the surface, is to direct your remedies to the congested organ, and subdue, by every means, the inordinate febrile accitement. If the brain be the organ invaded, the "cold dash" exerts the most decided influence upon the congested vessels, and allays, at the same time, inordinate febrile commotion.—

Children usually make great complaint on the first use of this remedy, but in a few hours, they enjoy it very much—frequently calling for its use. Many cases occurred in which the lungs and brain seemed to participate simultaneously, in the congestion; the state of the lungs was no objection to the use of the cold water. I was agreeably surprised the first time I used the dash, under these circumstances, to find that, although the cough and other signs of pulmonary irritation were very severe; it exerted no unfriendly influence over the pulmonary affection.

Antimonials and blisters are the last remedies which have been named; of their use little need be said. When febrile excitement was very considerable, and there was no particular necessity for the use of active evacuants, antimonials, given in sub-emetic doses, were found to allay fever, and frequently operate kindly upon the skin. Blisters were not used in the early part of the disease; there were two indications which they were intended to fulfil,—first, to equalize excitement without reference to local disease. When, in the course of the disease, the tendency to coldness of the extremities was obstinate, they were applied to the ankles and wrists, with their usual good effect. Secondly,—If after the necessary general depletion, local disease of any organ continued, blisters applied in the vicinity of such organ, were found to afford great relief. For this purpose they were applied to the nape of the neck, when the brain was the seat of disease; to the chest, in lung cases, and sometimes to the throat. When the disease fixed upon the lungs, there seemed to be greater necessity for their use;—these cases were usually protracted, and of their treatment blistering and cupping formed a very important part. In these cases, repetition of blisters frequently became necessary. In one case, I applied eleven, in succession, to different parts of the chest. If the first did not produce a crisis, it would dry immediately, perhaps in a few hours; and as their application scarcely ever failed to afford at least temporary relief, so the drying of them was always followed by an aggravation of the symptoms. Whenever, then, a blister was applied, a cessation of discharge from its surface was the signal for its re-application.

When the *lungs* were the seat of the disease, the cough, as I have said, was exceedingly troublesome. Various remedies were used for this, in addition to those already enumerated the compound syrup of squills (hive syrup) was much used and with good effect. Demulcents of various kinds were used; the following compound seemed to answer better than any thing else:

℞. Gum Arabic ℥ss.

Cream Tartar ʒij.

Dover's powder ℈j.

Water ℔ss.

To be given to a child, say two years old, in tablespoonful doses every hour or two. The dovers powder was sometimes omitted, and then the patient might use the cream tartar and gum water for its drink; but if not given in this way, the powder was usually given either alone or in conjuction with calomel, at least once a day; nothing seeming to exert so much influence over the cough as this, at the same time that it is not known to have had any bad effect. Acids, in this form of the disease, especially, seemed to exert considerable febrifuge influence. Some of the vegetable acids were usually given in the drinks, in every form of the disease. Digitalis should not be omitted in the catalogue of remedies for scarlet fever with pulmonary congestion; or rather for the sequelæ of the disease. In a few cases, I witnessed the happiest effect from this remedy, it was given in combination with the camphorated tincture of opium.

The *throat* seldom, if ever, escaped disease. Although in a few instances this affection threatened the life of the patient, in a vast majority of cases, it was matter of little comparative importance. Stimulating liniments applied to the external part, with gargles constituted the usual remedies. The common soap liniment, with spirits of turpentine, or when the inflammation of the fauces was very considerable, the tincture of cantharides was well calculated to produce the necessary amount of irritation. Vesication was occasionally produced in this way. As a gargle, the decoction of capsicum answered a very good purpose. I would not be misunderstood in reference to this symptom. I have said that in a majority of cases, it was matter of little comparative importance; yet, nevertheless, I am very sensible, that being a source of irritation, it tends materially to keep up the constitutional excitement, and may become the pivot upon which the fate of the patient may turn. I have frequently observed its influence upon the febrile commotion, and witnessed very sensible abatement of this, from slight vesication of the neck.

In the ulcerated state of the lining membrane of the mouth, heretofore described, a great variety of remedies were used, and it must be confessed, that local applications exerted but a feeble influence over it. In all these cases, the accompanying fever was of the low typhoid character. Although the fever in its early stage may have been inflammatory, yet so soon as this ulceration of the mouth occurred, its character changed: the pulse became weak, but frequent; patient irritable; no satisfactory examination of the inside of the mouth could be made; the soreness of this, and the lips, rendering it difficult to introduce even a spoon into the mouth. This condition of the mucous membrane of the mouth usually came on very suddenly; it would seem as if one of the effects of the disease, in these cases was to impair and almost destroy the vitality of this membrane. That the mucous tissue of the alimentary canal participated in this disorder, there could scarcely be a doubt. The remedies, so far as remedies could be said to exert any influence over it, were alkaline medicines, very much diluted, and quinine; this last given in full doses two or three times a day, or more frequently, assisted by gargles. I thought I witnessed more advantages from a strong decoction of Peruvian bark, acidulated with the marine acid, than any other gargle. But until the powers of the system were in some degree restored, no improvement in the condition of the mouth could be expected.

Is the disease contagious? This question is usually answered in the affirmative. Upon this subject the late epidemic here (for such it doubtless was) has, perhaps shed no new light. An advocate for either its contagious or non-contagious character, would not, I think, have been induced to change his views, but each might have collected many facts tending strongly to sustain his own favorite doctrine. If scarlet fever be contagious, it is not governed by the same laws that govern small pox, meazles, and some other diseases, the contagiousness of which is universally admitted. It seems to have all the attributes of an epidemic, and yet there are facts connected with its prevalence, which would incline us to suspect, very strongly, that it does possess, in some cases, and under certain circumstances, the power of propagating itself.

Case 1st,—January 20th, 1832. Little girl, daughter of J. P., 3 years old, attacked yesterday evening with vomiting, succeeded by pain in the head, and fever; these continued all night, slept none. When I visited this child at eleven A. M. she was comatose, pupils dilated and scarcely sensible to light; pulse 150, but compressible; rather weak; tongue furred; papillæ elevated and red, eyes suffused, skin dry and hot; fauces deep red, with ulcers on tonsils. Patient so insensible that it was with much difficulty we could induce her to swallow any thing, even water —feet cold.

Vs. 8ʒ. this induced great prostration and paleness, child had nearly fainted.

Gave an emetic of vin. ant. which operated well; this was followed by grs. x. of calomel. Sinapisms to the feet and ancles, the hair removed, and cold vinegar applied to the head. Soap liniment and turpentine [3 parts to 1] to the throat.

2 o'clock, P. M. Patient sensible, pain in head continues, pulse 160; has discharged bile by emesis, cathartic has not operated—feet warm.

4ʒ of blood drawn by cups from nape of neck and temples. ʒij. castor oil, with grs. iij. of calomel ordered every two hours, with injections, as often—cold to the head continued.

9 P. M. Medicine has operated very imperfectly; symptoms the same. 4ʒ of blood drawn by cups as before; magnesia is substituted for oil. Cold continued to the head, and capsicum gargle.

January 21st, 7. A. M. During the night the cathartic has operated freely, large quantities of consistent stools of a dark green color have been passed. Child much better, pulse 120 and soft, tongue cleaning. She behaves as if intoxicated, though when spoken to, is perfectly rational.

In the afternoon, the eruption appeared, but no further attention was necessary, than to keep the bowels open with castor oil, and the occasional application of cold to the head. Recovered.

Case 2.—March 28th. Little girl aged 4 years, daughter of J. D., attacked with vomiting at 3 o'clock this morning; visited her at 9 A. M.; skin dry and very warm; pain in the head; pulse 160 but soft; tongue white; papillæ slightly elevated; fauces red; and tonsils much swollen; eyes red; feet warm.

℞. Emetic of tart. antimoni. to be followed by grs. xij. of calomel. Cold dash to be used after the operation of the emetic, the hair being removed by scissors. Liniment to the throat, with gargle of decoction of capsicum.

12 o'clock, noon. Emetic has operated well. Calomel but just given; pulse 165 and hard; papillæ of the tongue more elevated; complains of much head-ache; eyes still red; feet warm. Regretted that I had not drawn blood—the cold dash has been used for more than an hour.

Vs. 10ʒ. castor oil to be taken every 2 hours, with 3 grs. of calomel, and cold dash repeated every few minutes, or as often as the head becomes warm.

5 P. M. Medicine has operated well; evacuations consistent; nearly black, with green tinge; pulse 150; some appearance of eruption. Cold dash continued; no medicine.

10 P. M. Eyes red; pulse 150; tongue unchanged; tonsils shew some ulcerated specks; fauces very red; skin dry; eruption extending itself very slowly—head easy.

℞. Calomel grs. 5. with ʒj. calcined magnesia every three hours. Dash continued.

29th, 8 o'clock, A. M. Medicine has not operated to any good purpose; spent a restless night, but no headache; pulse 140; eruption general, and of a scarlet redness.

℞. Calomel grs. xx.
Croton oil gtts. vj.
Simple syrup ℥j.

Mix and give a teaspoonful every two hours, and if necessary, ℥ss. castor oil at 2 P. M. Dash discontinued, but cloths dipped in ice water kept constantly to the head.

5 o'clock, P. M. Medicine has produced free discharges of dark consistent matter from the bowels. Skin cooler and soft; pulse 135; tongue cleaning at tip and sides; papillæ prominent; eruption general. Discontinued all medicine.

9 P. M. Patient doing well. Ordered ten grains of calomel, to be followed by castor oil or magnesia.

The recovery was rapid, nothing more being done than to keep the bowels open, which was easily effected.

In this case, the cold dash seemed to supercede the use of topical bleeding, and effectually to control the vascular action of the brain. The neglect of bleeding at my first visit had like to have cost my patient her life.

Case 3.—May 25th. B. T aged 7 years, was attacked about midnight with the usual symptoms. I saw him at 10 A. M.; pulse 160; complains of head; has vomited frequently; tongue white; papillæ distinct and prominent. Skin hot and dry; fauces and tonsils slightly inflamed; feet warm. I was making arrangements to bleed my patient, when I was required suddenly, to visit another patient, whose case would admit of no delay, and I thought perhaps it might be safely omitted; I therefore ordered an emetic of tart. ant. to be followed by 12 grs. of calomel, the hair to be removed, and the head kept wet with cold vinegar.

At 2 P. M. I was called in great haste—patient insensible; talks incoherently; bathed in a general and profuse perspiration; the whole body cold, except the head; no pulse to be felt at the wrists or ankles; pupils of the eyes much dilated, scarcely moving on the admission of light. He was immediately placed in water at about the temperature of 120°; kept there for five minutes; and on his removal, sinapisms were applied over the region of the stomach and to the extremities, and cups applied to each temple; by which nearly 6 ounces of blood were daawn. I remained two hours with my patient, and when the mustard induced redness on one part, it was changed to another. The cold dash was used immediately after the cups were removed, and repeated every few minutes during the time. At length he was able to swallow drinks in small quantities. Whiskey and capsicum tea were given in such quantities as he could be induced to swallow. After he had swallowed perhaps half a pint of the latter, with some two or three ounces of the former, he vomited, which was soon succeeded by return of heat to the surface, and subsidence of the perspiration. At 4, I left him, with instructions to continue the dash and sinapisms; was called again at 5; found the little fellow in convulsions, which had already lasted near half an hour. I re-applied the cups to the temples and nape of the neck, and in half an hour perhaps succeeded in obtaining 5 or 6 ounces of blood—the dash used every few minutes while this was doing. The spasms abated, and vomiting returned,—when he could be induced to swallow, the same drink was given; but the vomiting recurring every few minutes, nothing would remain on the stomach. At length, about 10 o'clock, P. M. his skin became generally warm, with gentle perspiration, vomiting ceased. 15 grains of calomel were given—cold dash continued, with external warmth to feet.

12 at night. Has not vomited since ten—pulse at the wrist can be perceived. No other particular change; he lies easy.

℥iv. of blood drawn from the temples, and the following medicine ordered:

℞. Calomel grs. xxv.
Croton oil gtts. viij.
Simple syrup ℥i.

Mix. Give a teaspoonful every two hours, ℥ss. Ol. Ricini at 1 o'clock, to be followed by purgative enemata. Dash continued.

26th, 6 A. M. Patient better; cathartic has operated well, dejections thick and of a dark green hue; patient quite sensible; pulse merely perceptible at wrist; extremities blistered by the mustard; epigastrium almost blistered. The body is almost covered by a very perfect scarlet eruption; child slept some in the last part of the night; complains of nothing but his blisters.

Cold sponging to the head, substituted for the dash. Omit medicine.

12 o'clock, noon. Pulse very perceptible at the wrist and ancles; cannot be counted, owing to its vibratory character; should suppose it is not less than 180; patient sensible, and complains of headache; eruption very general.

Give the croton mixture every 3 hours and a tablespoonful of castor oil in each interval, with injections. Continue cold to the head.

10 P. M. Medicine has operated imperfectly; skin hot and dry; eyes red; pulse much as before, only that there is a degree of resistance perceptible upon pressure.

Vs. 8℥ and 3℥ drawn from nape of neck by cups. Medicine continued, with the exception of the oil, for which senna tea is substituted. Injections every two hours. Resume cold dash.

27th, 6 A. M. Medicine has operated abundantly; dejections black; child every way better. From this time, nothing was done, except to see that his bowels were kept open; unless that cold to his head was used during this day. His recovery was rapid.

This case is highly interesting. It is one of those occasionally met with, not only in scarlet fever, but other congestive diseases, in which the physician must deplete and stimulate at the same time. In this case, the vessels of the brain were so injected with blood, and, as a consequence, so much enlarged, that they produced pressure upon the origin of the nerves, which, in one or two hours, probably, would have destroyed the life of the patient.

Post Mortem Examination of the body of a child three years and four months old, which died of scarlet fever, 36 hours after the attack.

January 8th, 1832. Twenty-one hours after the attack, in presence of Drs. Leslie, Dickson, Andrews, and Mairs, I proceeded to examine the body.

External Appearance.

Great discoloration of the skin of the back and most dependent parts of the body.

Internal Appearances.

*Abdomen.* On opening this, the bowels were found considerably distended with air, and containing a few worms. The gall bladder very much distended, and some yellow bile on the neighboring parts, as if it had escaped from the bladder. On pressing this, it was found impracticable to force the contents of the bladder into the bowel. Before opening the gall bladder, the stomach was removed and found to contain about 12 ounces of dark green fluid, which emitted no particular odor. The villous coat of the stomach of the natural firmness, but in many parts exhibited reddish patches: the peritoneum was streaked with red vessels. On opening the duodenum, a probe was passed from it into the ductus cholidochus, as far as the junction of the hepatic with the cystic duct, but was not easily passed into the gall bladder. The gall bladder was found, on opening it, to be full of black bile, having a shade of green, scarcely to be recognized. Nothing remarkable in the appearance of the liver; the pancreas, rather large and dense. Nothing here could account for the sudden death; I proceeded to examine the

*Fauces.* Left tonsil of the size of a hickory nut, and contained a small ulcerated cavity: right tonsil natural—papillæ on the back part of the tongue much elevated. On the posterior part of the pharynx, several ulcerated specks, some as large as half a grain of wheat. Descending the trachea, there was some redness for one and a half or two inches, after which the appearance was natural.

*Chest.* Pleura costalis exhibits slight vascular engorgement; the posterior part of the lungs, contains extravasated blood, supposed to depend upon the position of the body since death. No other mark of disease.

*Brain.* On removing the upper part of the cranium, the the cause of death was apparent in a moment, the veins of the brain, and sinuses of the dura mater were full of blood, as if violently injected; the vessels of the pia mater were swelled to four times their natural diameter, and so numerous, that the appearance of the brain was altogether unnatural. The child had died of apoplexy: In other words, although it is proper to say, that the disease was scarlet fever, yet the immediate cause of death was the vast amount of blood in the vessels of the brain, which, pressing upon the substance, had extinguished life. The same vascular injection pervaded the entire substance of the brain, including the medulla oblongata; the spinal marrow was not examined, but was supposed to be in a similar state.

